# Surgical Decision-Making for Temporal Lobe Epilepsy: Patient Experiences of the Informed Consent Process

**DOI:** 10.3389/fneur.2021.780306

**Published:** 2021-12-08

**Authors:** Sandra R. Dewar, Huibrie C. Pieters, Itzhak Fried

**Affiliations:** ^1^Center of Nursing Excellence, UCLA Health, University of California, Los Angeles, Los Angeles, CA, United States; ^2^School of Nursing, University of California, Los Angeles, Los Angeles, CA, United States; ^3^Department of Neurosurgery, University of California, Los Angeles, Los Angeles, CA, United States

**Keywords:** decision-making, epilepsy surgery, informed consent, thematic analysis, qualitative research, patient experiences, surgical risks

## Abstract

**Background:** Surgical resection is frequently the recommended treatment for drug-resistant temporal lobe epilepsy (TLE), yet many factors play a role in patients' perceptions of brain surgery that ultimately impact decision-making. The purpose of the current study was to explore how people with epilepsy, in their own words, experienced the overall process of consenting to surgery for drug-resistant TLE.

**Methods and Materials:** Data was drawn from in-person, semi-structured interviews of 19 adults with drug-resistant TLE eligible to undergo epilepsy surgery. A systematic thematic analysis was performed to code, sort and compare participant responses. The mean age of these 12 (63%) women and seven (37%) men was 37.6 years (18–68 years), with average duration of epilepsy of 13 years (2–30 years).

**Results:** Meeting the neurosurgeon and consenting to surgery represented an important treatment milestone across a prolonged treatment trajectory. Four themes were identified: (1) Understanding the language of risk; (2) Overcoming risk; (3) Family-centered, shared decision-making, and (4) Building decisional-confidence.

**Conclusion:** Despite living with the restrictions of chronic uncontrolled seizures, considering an elective brain procedure raised unique and complex questions. Personal beliefs and expectations related to treatment outcomes influenced how the consent process was ultimately experienced. Decisions to pursue surgery had frequently been made ahead of meeting the surgeon, with many describing the act of signing as personally empowering. Overall, satisfaction was expressed with the information provided during the surgical visit, despite later inaccurate recall of the facts. These findings support the resultant recommendation that the practice of informed consent be conceptualized as a systematic, structured interdisciplinary process which occurs over time and encompasses three stages: preparation, signing and follow-up after signing.

## Introduction

The advantages of epilepsy surgery for seizure control have been established by Class 1 evidence (1, 2). However, electing to under go an irreversible brain procedure represents a complex decisional process for both people with epilepsy (PWE) and clinicians, that includes weighing surgical risks against expectations for seizure freedom and a hopeful future (3). Even in well-selected candidates, and despite illness severity, firmly held beliefs especially fear, influence whether patients will choose or defer surgery (4, 5). Although the risks of permanent, noticeable neurological deficits due to surgery are small (6), physician and patient misperceptions of surgical risk remain a significant barrier to an effective treatment (7, 8).

In general, a central goal of informed consent is to inform and manage risk such that patients feel they have sufficient information about a planned procedure and the alternatives, to make a rational choice (9). Risk management is a process that takes place over time (10), incorporates the contributions of multidisciplinary team members and is foundational to building trust (11, 12). How surgical risks are presented and understood have implications for approaches to providing information about surgery and ultimately signing (or not) an informed consent (12, 13).

Specific to epilepsy surgery, preoperative counseling includes weighing up the substantial risks for morbidity and mortality of uncontrolled seizures vs. the potential risks of the operation. Complication rates associated with epilepsy surgery are reported as “low” and mostly minor and temporary (6). Counseling for epilepsy surgery should be individually tailored to balance the possibilities of seizure-freedom after surgery against potential procedure related cognitive losses (14). The impact of surgery on memory is always a consideration in TLE, leading authors to suggest that memory decline should be viewed as a cost of surgery and not necessarily a surgical risk (14).

The goal of epilepsy surgery is to achieve seizure freedom. However, a potentially life-changing shift from chronic disability to sudden wellness evokes unique psychological and philosophical adjustments described as the concept of ‘burden of normality' (15). In a substantial body of work, Wilson and colleagues illustrate the concept in a theoretical framework comprising the broad, interrelated psychosocial features that shape patient identity and family behavior. This framework that conceptualizes the reversal of disability has significant application to surgical outcomes (16). The identification of key features are frequently neglected by questionnaires related to quality of life (15). Consequently, the role of the epilepsy team in comprehensive patient evaluation is underscored, with specific emphasis on the potential of specialized epilepsy nurses for promoting individualized care and shared decision-making.

Although the complexity of decision-making in epilepsy surgery has been described (14, 17, 18), there appears to be a dearth of studies in the epilepsy literature dedicated to the process of obtaining the informed surgical consent. Signing an informed consent does not guarantee the understanding of information provided, as shown in studies of informed consent in clinical trials research (19) and a qualitative research study of patients who had consented for general surgical treatment (20). What was understood and remembered was mostly subjective and influenced by emotional responses (21). To improve communication, supplemental, high-quality decision-aids have been shown through randomized control trials to be effective in supporting understanding and recall (21, 22). Decision-making shared between patient and physician, actualizes the concept of informed consent (12). Such shared decision-making is especially important when treatment outcomes are uncertain (12), and therefore holds specific relevance for decisions about epilepsy surgery. Underutilization of effective and safe surgical therapy for epilepsy is longstanding and vexing (23, 24). Furthermore, the recently documented decline in number of open resective epilepsy surgeries performed (8), co-incides with the advent of new surgical therapies, such as responsive neurostimulation and stereotactic thermal ablation. In light of expanded therapies, understanding key aspects of surgical decision-making naturally takes on heightened importance.

Conclusions from our larger study of patient perceptions of risks and benefits (3), were that an elective brain procedure for a chronic and frequently life-long condition, raised uniquely complex questions for PWE and their families when surgery was presented as an option. As part of the parent study, the purpose here was to explore how people with pharmaco-resistant TLE experienced the process of signing the informed consent for epilepsy surgery. As an understudied aspect of treatment, exploring personal experiences of this consenting process may offer a new angle on how to overcome underutilization of epilepsy surgery.

## Materials and Methods

The techniques of constructivist grounded theory guided the initial design of this qualitative study, the data collection, and initial analysis. The philosophical cornerstones of grounded theory included paving the way to understanding how study participants construct what is meaningful to them and how they act. This methodology was selected as the best way to explain how knowledge was perceived, internalized and recalled (25). We utilized the flexibility of thematic analysis to present the findings (26).

### Setting and Background

Data for this manuscript originated from a parent qualitative study (*N* = 35) where the contextual basis of decision-making centered on how treatment risks and benefits were described (3). In the present study, we focus on the subgroup of 19 participants who had signed an informed consent to undergo a craniotomy and surgical resection of epileptogenic tissue. Study participants were adults seeking treatment for uncontrolled temporal lobe seizures at a leading, academic epilepsy center in the United States. Before they signed the informed consent, the first author, a clinical nurse specialist, invited eligible patients on the surgical waiting list to participate in a semi-structured interview about their understanding of treatment options including surgery. All invited patients in this convenience sample agreed to participate.

*Standard consent process*. At the time the informed consent was obtained, patients were typically meeting and receiving information about the surgery from the neurosurgeon (IF) for the first time and simultaneously signing a surgical consent form should they wish to proceed. The clinical nurse specialist (SD), who was known to the patients, was present to actively contribute to the consent process by articulating key elements of the clinical history relevant to individual surgical risks and to clarify explanations for the patient, family members and surgeon.

The consent form was a standard document used for surgical procedures at our medical center. Upon signing, the patient agreed that a description of the surgical procedure was provided, the reasons for the surgery had been given, and the risks, benefits and treatment alternatives have all been explained. Patients and any caregivers present were given the opportunity to ask questions about the procedure, the recovery process, and the benefits of surgery. The name of the procedure was repeated several times during the consultation and a copy of the signed consent form was provided to the patient. Five (26%) participants received surgery within a week of signing the informed consent. Of the remaining 14, 12 (63%) participants underwent surgery several weeks later while 2 (11%) eventually opted not to undergo surgery.

### Participant Selection, Study Procedure and Data Collection

Institutional Review Board approval was obtained prior to recruitment. Participants were English speaking adults with drug-resistant temporal lobe epilepsy, who were legally able to sign their own surgical consent. Patients who had undergone a previous brain resection were excluded to avoid the potential bias that a past experience of signing an informed consent may bring. An in-person research consent was obtained and a time convenient to the participant was scheduled for the interview once the surgical consent had been signed. All interviews were conducted one-on-one in a comfortable, private room in the clinic, by an experienced, female qualitative researcher with a background in neuropsychology, and who was not part of the clinical team. A semi-structured script provided a flexible guide for data collection. Selected questions from the guide are provided in Table 1. Although seven (37%) participants chose to be interviewed on the same day as meeting the surgeon, the interviews were conducted an average of 31.2 days after the consent had been signed.These audio-recorded interviews lasted an average of 105.7 min each (range: 54–147 min). Participants received a honorarium of $50 cash on completion of the interview as a token of appreciation for their time. Demographic and clinical characteristics of the sample were derived from the medical records (Table 2). The verbal comprehension index (VCI) was obtained from the standardized neuropsychological battery derived from the Weschler Adult Intelligence Scale (WAIS) version 4. Neuropsychological testing conducted by a licensed psychologist is an essential component of an epilepsy surgery evaluation at our medical center.

**Table 1:** Examples of conversational interview questions.

**Setting the scene**
You have come to an important time in the treatment of your epilepsy. Can you tell me about the surgery you are having? Part of signing a consent for surgery is to hear about the risks of the procedure, how do you understand the risks of the surgery? Follow-up: How do you think surgery for your epilepsy will be of benefit to you? The possibility that surgery will stop the seizures was given to you as a percentage. When it comes to understanding this number, what does it mean to you to hear from the surgeon that you have a xx% (% specific to the individual) chance to be seizure-free after surgery? Follow-up: How is it helpful to hear about seizure freedom as a percentage? Once you had signed the consent and agreed to the procedure, how did that feel for you? Follow-up: Can you describe any specific thoughts that crossed your mind? What was it about talking to the surgeon that gave you confidence to go ahead with surgery? Can you tell me what has helped you to be certain that the benefits of surgery outweigh the risks? How did discussions with your family help, or not help you to make the decision to have surgery? Can you tell me about the specific questions you or your family had for the surgeon? Follow-up: Is there anything that is worrisome about the surgery now that you have had the chance to talk with the surgeon? If so, can you tell me about these concerns? How does having a team involved in your epilepsy care help you to feel positive about the surgical decision? On a scale of one to ten, how satisfied are you that you had all the information you needed to make this important decision? Ten is totally satisfied. One is totally not satisfied. How might it have been helpful for you to talk to someone who had undergone epilepsy surgery?

**Table 2:** Sample characteristics (*N* = 19).

**Clinical**	
Age at interview: mean (range)	37.6 (18–68)
Duration of interviews (range)	105.7 mins (54–147)
^*^Age at seizure onset in years: mean (range)	24.4 (3–60)
^*^Duration of epilepsy in years: mean (range)	13 (2–30)
Days from consent to interview	31.2 (0–168)
Days from consent to surgery (*n* = 16)	57.5 (1–169)
Side of proposed surgery	
Right	12 (63)
Left	7 (37)
	***n*** **(%)**
Gender	
Men	7 (37)
Women	12 (63)
^*^Number of anti-seizure drugs at time of consent	
1	1 (5)
2	7 (37)
3 or more	11 (58)
^**^Verbal Comprehension Index (VCI)	
Borderline (70–79)	2 (11)
Low average (80–89)	1 (5)
Average (90–109)	12 (63)
High average (110–119)	1 (5)
Superior (120–129)	2 (11)
Very superior (130 and over)	1 (5)
**Demographic**	
Ethnicity	
Caucasian	13 (68)
Hispanic	3 (16)
African American	2 (11)
Asian	1 (5)
Marital status	
Never married	7 (37)
Married	10 (52)
Divorced or separated	2 (11)
Living situation	
With spouse	6 (31)
With spouse and children	3 (16)
With children (no spouse)	2 (11)
With parents	6 (31)
With roommate	2 (11)
Highest level of education	
≤ High school	7 (37)
Some college	5 (26)
Completed undergraduate degree	5 (26)
Completed graduate degree	2 (11)
Gainfully employed (full, part or self)	9 (47.5)
Unemployed	9 (47.5)
Retired	1 (5)
^*^Health insurance provider	
Private (through own or parent employment)	14 (77)
State Health	5 (23)

* *Source: Medical records.*

** *The Verbal Comprehension Index (VCI) measures verbal reasoning ability. This includes the ability to listen to a question and create a verbal response that expresses the person's thoughts. The index is a composite score of three tasks that measure word similarities, vocabulary and comprehension*.

Extensive analytic memos were written after each interview to describe analytic hunches, identify commonalties and differences, and inform potential new questions to promote deeper descriptions in future interviews. In addition, observational memos were written by the clinical nurse specialist shortly after the surgical consent was obtained to record her observations of the process. As part of the study, and since she played an active role in this aspect of treatment, the CNS paid special attention to how the information was presented, and observed the emotional responses of the patients and family members who were present.

### Data Analysis

Interview scripts were transcribed verbatim by a professional transcriptionist, checked for accuracy and de-identified. Data analysis by the first and second authors followed the principles of a robust, thorough and systematic analysis based on the phases of coding described by Braun and Clarke (26). The data set for the parent study was coded independently by the analytic dyad (SD and HP). Codes were agreed upon, sorted and compared across participants to identify similarities and differences. Reflexive memos in qualitative research contain the clues to recurring ideas in the data and their relationships to one another (27). Our memos contributed to data interpretation, and supported the identification and development of themes. Regular meetings throughout the analytic process boosted interpretations that reflected the data. ATLAS.ti was used to manage and organize the codes and record the reflexive memos (28).

In keeping with the guidelines on data analysis of Braun and Clarke, the ongoing dialogue between the analytic dyad facilitated agreement upon four themes that accurately captured the essence of a rich data set (26). Theoretical saturation was reached once the themes with their properties and dimensions were determined to represent a logical, detailed and complete account of the data.

## Results

The sample characteristics, shown in Table 2 included 19 adults with a mean age of 37.6 years (18–68), and drug-resistant temporal lobe epilepsy for an average of 13 years (2–30). Of these 12 (63%) women and seven (37%) men, seizure onsets were localized to the right temporal in 12 (63%) cases. The study participants reported an average of 14.2 years (12–20) of education. Verbal comprehension scores obtained from neurocognitive testing fell within the low to high average range in 14 (74%) cases, two (10%) were within the borderline range and three (16%) were in the superior range. As a component of IQ, these scores reflect verbal reasoning ability, and include a person's ability for comprehension and verbal expression. Interviews were conducted an average of 31 days (0–168) after signing the consent with seven (37%) preferring to interview on the same day, shortly after meeting the surgeon. Surgery itself occurred an average of 57.5 days after consenting (*n* = 16) (Table 2). Excluded from the latter calculation were two patients who declined surgery and one whose surgery was unusually delayed for 355 days.

Meeting the surgeon and signing the consent form represented an important treatment milestone. Although the surgery followed an extensive evaluation process and was viewed as a positive step, the reality of removing brain tissue lay outside personal experience. Surgery signified an opportunity to achieve seizure freedom and lessen caregiver burden, but the risks of a surgical complication, albeit presented as “low” still meant that fear and uncertainty had to be overcome. A woman in her mid-thirties expressed the existential meaning of “cutting out a piece” of a vital organ and the courage it took to consider elective brain surgery saying:

When I was diagnosed with epilepsy nine years ago, I was totally against surgery because I was scared. It was something totally new, and it made me feel old. Now I'm not scared. I'm ready…Now it's time to move forward and do something.

Despite inherent unknowns, it was also apparent that decisions in favor of surgery were frequently made in advance of meeting the surgeon, and signing the IC was viewed as a simple formality. Yet, having signed the consent, becoming mentally prepared for surgery became all-consuming and was described as “the last thing on my mind when I go to bed, and the first thing on my mind when I wake up.”

Overall, four themes informed the experiences of signing the IC. The first, understanding the language of risk, included comprehension of the procedure and the surgical risks. The second encompassed how risk was overcome and benefits prioritized. Theme three revolved around the integral nature of family-centered decision-making and emphasized who ultimately owned the surgical decision. In the last theme, decisional confidence and satisfaction with information was captured. Illustrative quotes representing each theme are provided in Table 3.

**Table 3:** Illustrative quotes supporting the four themes and dimensions.

**Theme**	**Dimensions**	**Examples**
Theme 1: Understanding the language of risk	Recalling the brain procedure Explaining a visual field deficit	Remove the bad piece of whatever it is that's starting to get crushed. [P11] They're going to be operating on the right front side of my brain. And its corrective surgery. [P19] They would be opening my skull, taking out, if you will, a portion of my brain, putting my skull back together again, and there you are. [P02] I don't think it is a bacteria, but there is a bad spot in my brain…He (neurosurgeon) said he would cut a hole in my skull and go in and take some tissue out. I didn't want to do that because I didn't want that kind of invasive surgery. He said it [the lesion] was close to the surface and it wouldn't be harmful. It wasn't in the hippocampus or the cortex or any of that. [P05] I understand that they are going to be going in and removing a portion of my right temporal lobe as well as half of my hippocampus as well as half my amygdala. They are doing that because they do not know the absolute specific spot, but they have a really good general idea of the location…I understand that they're going to do a question mark-shaped incision on my head and that it will flap open and that they will do the surgery that way and that they will have to shave a portion. And then that's how they'll do the, uh, sewing back up. [P18] It's interesting that this condition is in the left temporal lobe, because apparently left temporal lobe is communication. However, I was able to pass the test for a radio license for emergency services. I'm 100% bilingual. So, it's pretty interesting how, because one side is jammed, the right side completely took over. That's a pretty interesting concept. [P06] He did tell me that there is a possibility of not being able to see from my left side but only on the top. And he said, “Well, we can turn our heads to be able to see. One way or another, you have to turn your head.” But that was the only risk. It does kind of scare me because I don't want to go blind, because right now I can see perfectly. [P12] There may be something small that might happen and create - like he told me about my eyes, my peripheral vision. I said, “Will that stop me from driving?” No, because you can just turn your head and see what you need to see. But I never thought that the brain surgery would even affect that. [P07] They said I could lose sight of the corner of my right eye. But that's not a big concern because I mean everything else is going to be fine. [P03]
	Procedural risks vs. benefits	[There is a] ten percent chance that something might go wrong, I think he says. But I've gotten that number out of my head already…I'm a very optimistic person. Ten percent is very low. [P08] Essentially, when I was given the percentages, it was the risks vs. the benefit. So, they told me - what did they say? It's like 70 or 80% chance that it would cure my seizures. Yeah. So, the benefits of it were really good, and there's a strong percentage of it…And then some of the risks, I was given really low percentages. I don't remember what they were exactly. [P09] Surgery could impart an increase in speech impediment, a decrease in mobility, a decrease in eye movement or being able to see lateral imaging. [P02]
	Contemplating percentages and	I am concerned about that 2% risk of complications. Any time you open a brain, you open a skull, and you start playing with the brain, there are certain unknown factors. [P02] A one-in-five chance doesn't seem as bad as 20% because you just hear the larger number [P01] The way I understood that was how like, she was saying, “It's not a 100% chance that they're going to stop when you have the surgery, but probably a 70%,” that it's usually a 70 or 80. And 70's pretty high, to me. And then like, it was an 80% that he was a good surgeon, like he does things very well. And that's pretty high as well. [P14] I'm like 99% sure that everything will be fine. The doctor did tell me it is like only 1% that anything [bad] will ever happen. That's with any surgery. [P15]
	The meaning of numeric probability	We have a 70% confidence that this is going to be successful. And that seems terrible. But I guess…with this kind of surgery 70% is decent. I'm sure it's being low-balled because they don't want to say, “We're 97% positive that this is going to be fine”, and then it's not. [P17] He had told me it was 60% that it could be seizure-free. I'm like, “Wow. That's pretty high.” And my husband liked that number. But when I called my mom and told her, she's like, “Oh. That's only 60% chance? I thought it was going to be higher.” And I'm like, “Okay. You just made me feel really good. [laughs] Why did I call you?” I think that 60% is a pretty good number, and I think surgery is worth a try. [P15] I was told - an 80% success rate with the surgery - I might be able to function as an average person later on down the line. Therefore, I might be able to drive. I might be able to be independent as a young adult and be able to be a winged pilot later down the road as a career. [P06] I was hoping that he [surgeon] would say, “Hey. Let's get it done.” And, at the same time, I don't expect like a miracle right away after the surgery. Like I said, I'm quite aware that it's not over yet. But when someone tells me 75%, then, to me, it's, of course, the other 25% - anything can happen. So, it's like, why not if it has been such a painful journey for ten years. [P16]
		I know for a fact that there's an 80% chance of being seizure-free with the chance of medication on the side, …which is excellent. And 80% is a high chance. Most that I have read on Facebook, were given 60% chances. So, with an 80% chance, that's an even higher chance that what I've read on most of those people that have had the surgery done. So, that's a high chance for me. And, umm, I like that. [chuckles] And then um - [pause] I got lost. [P04]
Theme 2: Overcoming risk	What it was like to sign	I had a lot more feelings of being alone within it [the decision itself]… I've never been unsure of the decision I was making… but I've been surprised at how emotional I have been. [P07] When I got home, [after signing the consent], it really sank in. My wife and I were pretty freaked out. We were both just really overwhelmed. [P09] I went through a period of mourning, shock and denial. I was just sobbing. It was such a difficult decision to make…[After signing] I don't feel like I can talk about much else but the surgery. [P18] I think nothing much [about signing]. I've signed so many consent forms, it's like, just another waiver. [P04]
Theme 3: Family centered decision	Taking ownership	Signing was a big weight off my mind…I'm doing this for myself, but I have my family who don't like to see me have seizures [P01] My concern is putting her [wife] at ease getting her past this anxiousness, getting her past the period of doubt, worrying about, are we doing the right thing. [P02] It was more his [husband] decision than mine, and I was ok with that. He said he wanted it to be completely my decision…I knew that if something went wrong, he'd be okay with that because it wasn't my decision completely. [P18] I'm done with this epilepsy. And [my mom's] like “Okay. Let's do it”. I don't think my family is doubtful about surgery. I think they're quite hopeful. [P19]
Theme 4: Building decisional confidence	Satisfaction with information Trusting the professional opinion Gaps	I'm very satisfied. I know that my satisfaction simply comes from being able to call (name of CNS) and (Assistant to neurosurgeon). That's where my satisfaction comes from. Because, if there's any question, I know at the drop of a hat I can make a phone call and talk to them. And I know they will answer their phones. And if they don't, I will get a call back. [P01] I did a lot of studying. I got online, and I went to a lot of different things on Facebook, looked up a lot of people that have had the surgery, read a bunch of things that people have - you know, their outlooks on it. I didn't so much put my own opinions in as much as I read what they had to say. [P04] The staff here explains it enough to where I'll understand. And I don't need to know everything about it because I probably won't retain all of it. [laughs] But they explain it enough so that I get a mental picture of it. [P19] With all the studies that I've done and everything - and, you know, I have a lot of faith in those studies just from being here - I think a ten. [P03] The confidence in the people I have spoken with, their body language, the way they come across, their knowledge of certain aspects of what's taking place, their history involved in this field, their academic achievements, their raising of pros and cons. And that's something I look toward in a person. If I'm going to put my hat in their corner, I want to know as much about them and their knowledge about what's going to happen and what could happen or what should happen as possible. And so far, I've had nothing but good things come my way with regard to this group of people. [P02] [Meeting a person who had undergone surgery] would help me kind of understand and expect, not just going in there like I'm blindfolded…And how was it for them too? And was it scary for them, or should I be not scared? I would've liked to have known that. But, unfortunately, I was never able to meet anybody with brain surgery. [P03] I'm a very visual person. If he had showed me [on the MRI] that one little dot that he was going to be removing, that would've helped me understand what he was going to be doing. [P05] I can see all this list of bad things that have happened or bad things that are possibilities. Have these actually happened? How many have this happened to? What are the percentages? I never got the sense that I even was given a percentage of how many people actually have dealt with side effects from this. So, I would like to have known more about that. So, yes, I felt like that's still being held back from me and that nobody really wants to tell you what the bad things are. [P18 ]

*P, Participant number*.

### Theme 1: Understanding the Language of Risk

#### Recalling the Brain Procedure

Participants' views of the brain as a vital organ influenced how surgical decisions were made. Such views included questioning whether seizures themselves could damage the brain, but also evoked images of the life-long implications should a surgical complication occur. Recall of the procedure consistently included locating the head shave above the ear, in the general region of the temporal lobe, and stating that the skin incision would take the form of a question mark. Although the scar would be behind the hairline, the size of the incision and the amount of tissue to be removed mattered. Participants commonly referred to a “small” incision or a “small” head shave, and resection of a “small” amount of tissue. To underscore a shared experience, participants spoke collectively about their understandings as illustrated in the words of one person who said of herself and her spouse, “We had a picture of ourselves having a dime-size taken out of my brain. By the time I was done talking to the surgeon, we realized we were talking about a much larger section.”

When asked what operation was to be done, participants had frequently forgotten the procedure name, and described the surgery in general terms such as, “lesion extraction,” “corrective,” or “minimally invasive?” In recounting the surgeon's explanation, one participant shared, “I didn't know all the words he was saying about the pieces of the brain, but I know that it's up here [points to left side of head].” Although the surgeon stated that surgery was to be performed on the anterior or front part of the temporal lobe, some participants explained that surgery would be on the right or left “front side”, others referred simply to “removing the part that's causing the seizures”. Seven (37%) participants clearly named the temporal lobe as the site of surgery, and two were also able to correctly name the mesial structures, i.e., the amygdala and hippocampus as implicated in the seizure network.

The specialized memory functions of right and left temporal lobe structures were explained by the surgeon, nevertheless participants' concerns about post-operative changes in memory tended to center around losing memory for important past events, or fears of forgetting loved ones. Especially in the language dominant group (*n* = 7), word-finding deficits were understood to imply a communication difficulty with one participant imagining that “loss of speech” would mean he would need to relearn language as in childhood. A participant who required a left-sided antero-mesial temporal lobe resection explained her understanding of the brain region and what it meant to her to have surgery in this part, saying that the surgeon “doesn't want to get close to my speech and memory section because they're very strong…, and he does not want to interfere with a part of who I am.” Two participants feared language deficits despite the plan for surgery on the side contralateral to language localization. Concerns about memory loss were expressed across participants especially since poor memory was a pre-existing challenge. Yet for some participants, stopping the seizures raised hopes that memory would improve after surgery to potentially improve learning ability.

The potential for a visual field deficit, was consistently remembered however this deficit was sometimes understood to imply vision loss, blindness, or restricted eye movements. Although participants were uncertain about what a visual deficit meant, the relevance of the deficit was openly dismissed, and expressed as “not a big deal.”

Dying from the procedure was a risk that was always mentioned by the surgeon, however, participants did not initiate further discussion about this possibility. In contrast, fear of dying was expressed during the interviews, with many participants foreseeing deep family grief should death occur. Although viewed as improbable, the possibility of dying was seen as part of the reality of surgery. Some participants spoke about the potential for dying from seizures, but most also thought this was unlikely to happen to them. Participants rationalized that living with post-operative motor or language impairments, might be worse than dying from the procedure. The possibility of weakness or paralysis due to stroke meant “having useless body parts” and being functionally worse off.

#### Understanding Percentages

Taking into account individual elements of the clinical presentation, two percentages are typically presented during the consent process: the risk of procedural complications and side-effects, and an estimate of seizure-free outcome. The possibility of serious procedural complications for temporal lobe surgery were usually quoted as less than one percent with participants in the present sample given a likely probability of seizure freedom ranging between 60–80%. While participants were generally concerned about surgery on the brain, hesitancy appeared greatest when they, or the family, focused specifically on the possibility of procedural complications rather than the benefits of seizure freedom. In contrast, when the benefits of seizure freedom were uppermost in people's minds, the surgical decision appeared to be made with less hesitation. Anticipating 80% seizure freedom was associated with “cure”, by several participants. One person was disappointed in an estimated 70% seizure-free rate and felt the number was purposefully “low-balled”.

The two percentages (procedural risks and estimated seizure-free outcome) were frequently mis-remembered. Some participants spontaneously explained that risks were quickly forgotten, as they preferred to focus on the belief that everything would go well. Despite incorrectly remembering the risks to be 10–15%, the over-riding take-home message was that surgical risks are “low” or “very, very rare.” Regarding the second percentage routinely discussed during consenting, there was unanimous acceptance that surgery would not offer 100% guarantee of seizure freedom, yet participants were cautiously optimistic. One participant said she was quoted a 25% possibility of seizure freedom which she determined was acceptable to her and represented a “big chance” of control. Regardless of the percentage quoted, participants, in their own minds, anticipated better outcomes. Although most participants were happy to hear percentages, one person preferred to hear a 1-in-5 chance vs. 20% (ie. a percentage), because “the numbers are smaller.”

Overall, hearing the numbers was helpful in decision-making even when the percentages were recalled incorrectly. Clearly, the number itself held meaning beyond the numeric value. As such, the percentage was not a defining entity, but represented a fluid and hopeful measure, especially when compared to past experiences with ineffective anti-seizure drugs.

### Theme 2: Overcoming Risk

Participants who expressed little hesitation to undergo surgery tended to minimize procedural risks and focused on the benefits of a positive outcome, that included coming off anti-seizure drugs, driving and “fixing an epileptic self.” A young woman who weighed the benefits of surgery as 99.9% greater than the risks said, “I'm not worried about the risks. Thinking about the pros and cons, will get me nowhere.” Others also admitted to quickly dismissing the surgical risks, preferring to focus on an overall positive outcome as exemplified by the participants who rationalized that a 1–2% risk meant there was a 98% chance of a good outcome leaving “the 2% to be ignored.”

Across the sample, individuals reasoned that their quality of life needed to improve and that, without surgery, seizures and memory were likely to worsen. Perceptions of an optimistic future were guided by faith, including beliefs that surgery was in God's greater plan. When reflecting on their drug-resistant disease, many reasoned they had little choice, and nothing to lose but to go ahead with surgery.

Trust in the surgeon's skill was necessary to overcome a natural fear of brain surgery. When weighed against hard-won personal accomplishments such as academic or work achievements despite epilepsy, participants feared a changed or diminshed self after surgery. While some did not consider themselves to be limited by the seizures, a negative surgical outcome would be life-limiting. Participants in the current sample deliberately identified non-verbal cues that projected the surgeon's considerable experience and his confidence regarding a positive outcome. In addition, participants looked to family members who were present during the clinic visit to affirm trust. Faith in the institution and the team, especially the neurologist were spontaneously and frequently expressed as playing key roles in accepting the surgical option.

#### What it Was Like to Sign

High risks were naturally associated with brain surgery, making signing the consent an overwhelming experience for some. These emotional responses to knowledge about the surgical risks came as a surprise to the individuals and differed widely across the sample ranging from deep relief and excitement, to feeling overwhelmed and anxious. Excitement centered around seeing surgery as a proactive step after many years of disappointment with medicines. Visceral responses such as losing appetite and crying were reported to last for many days after signing. For some, the act of signing triggered self-reflective thoughts around what was important in life. For younger participants, the act of signing symbolized the assuming of an adult responsibility. While some participants expressed resignation about signing the consent, others viewed the IC as routine, non-binding, and simply a legal formality. A participant with a superior verbal comprehension score was critical that the focus of the clinical encounter was on surgical risks, and expressed her preference for more information on the benefits of surgery for daily living and life-style adjustment. Overall, surgical therapy was viewed with optimism especially when weighed against recurring disappointments of drug failure.

### Theme 3: Family-Centered, Shared Decision-Making

All participants ultimately took ownership of the final decision to undergo surgery, but family opinions were both powerful motivators and barriers. Until this point, family members had initiated and supported specialized epilepsy care, but embracing the decision to undergo brain surgery was far more complex. It was apparent that patients and families were observed to process fear of brain surgery in different ways, and to view the need for epilepsy surgery and the inherent risks differently. There were many advantages for family caregivers to be present for the surgical explanation as discussions with the surgeon could be heard first-hand by all concerned. Three participants did not have family members present. For the majority of participants, questions raised by family members contributed positively to the decisional process, to increase confidence in proceeding with surgery. Neverthess, the inherent ambiguities of surgical decision-making were exemplified when a middle-aged, professional man with right TLE reflected upon his family's views of surgery:

“My wife is supportive of the surgery. She feels that there may be benefits. She's also as realistic, as I am, about not seeing it as a panacea or a cure-all but that there are, hopefully, some benefits to doing so. So, she's supportive of it. I don't think my sons have a clear understanding of the course of recovery.”

Owning the decision to have surgery meant taking personal responsibility for the decision and the outcome and appeared to be a way to protect the family should complications occur. The focus for a mother whose seizures began at age 29 years, “was just to survive the surgery” and later to “deal with whether the epilepsy was cured.”

The words of one teenager echoed those of other young people who said, “It's my brain, it's my body?. I'm no longer underage. I feel it's my decision to have surgery because I've gone through the seizures.” However, for one person a shared decision, implied sharing the responsibility for the decision should something go wrong. For most participants, decision-making included the family, and sometimes friends, but the decision took time and was clearly not straightforward. Ultimately, individuals declared that the time was right. There was little to lose by moving forward with a procedure, and participants were motivated by the need to reverse disability.

### Theme 4: Building Decisional Confidence

Consenting for epilepsy surgery was the last formal step in a complex treatment process. The consent was viewed as a “formality” by some, and by others as a “symbol of reality.” Agreeing to surgery was based upon an active process of gathering information and building confidence in the decision. Participants spontaneously mentioned their observations of non-verbal cues. Sitting and talking, and making good eye contact were valued and identified as central to communicating the surgeon's confidence in the treatment approach.

The opinions of caregivers mattered. The positive affirmation of caregivers supported the surgical decision. Even when the clinics were perceived to be busy or visits felt rushed, participants rationalized that they were getting the best possible care. It was also apparent that participants and caregivers needed time after signing the consent to review their understanding and expectations, and in some cases to talk to other patients.

“It's calming for me to see that other people have gone through what I'm getting ready to go through and that they come out of it okay, and that they're not having seizures or they're having a lot fewer seizures. And they're on a lot less medication; and they're only having problems with wordplay; and they're still able to walk, talk, function, and, you know, some of the things that I'm curious and afraid of.”

Confidence was drawn from a comprehensive work-up that had been completed at a specialized epilepsy center, where the treatment plan was based on an interdisciplinary consensus. As one young man said, “a group of specialists makes the experience better. Having a team that is rooting for your wellbeing gives hope.”

#### Satisfaction With Information

When participants were asked to rate their confidence about how well-informed they felt to make the surgical decision, the median score on a scale of 1–10 was eight. One person said she was unable to provide a rating and was not included in the calculation. Although five (30%) participants were perfectly confident that they had all the information they needed, common reasons for less than 100% certainty included poor memory, and not knowing what questions to ask due to unfamiliarity with brain surgery. Lacking expertise in epilepsy surgery, in addition to uncertain expectations reinforced the need for implicit trust in the medical team. One participant (who came from a medical background) felt that risks were “downplayed”; that numbers who had died or suffered complications may have been purposefully withheld. Another felt that 100% certainty was not possible secondary to poor memory. Many people embraced self-education, but some did not know where to look for reliable information. Participants sought out and valued clarification of the surgical explanation from an experienced epilepsy nurse specialist.

## Discussion

The aim of this descriptive, qualitative study was to explain what it was like for a sample of patients with drug-resistant epilepsy to experience the informed consent process for anterior temporal lobectomy. Drug-resistant epilepsy constitutes an inherently unclear illness course but agreeing to an elective *brain* procedure introduces new levels of uncertainty. Despite intrinsic uncertainties, the act of signing a surgical consent was often experienced as empowering in the context of a disempowering condition. Consenting symbolized a personal turning point that represented hope for an independent future. While opinions of family members played a role in treatment decisions, the participants unanimously assumed ownership of the surgical decision. Although detailed recall of the procedure and the risks were often incomplete or inaccurate, participants accepted that surgery was the best therapeutic option, and that risks while small, were not zero.

Distinct challenges confront both the treatment team and the patients when considering epilepsy surgery. While the surgical procedure is guided by underlying pathology it is tailored to the individual talents and lifegoals of the patient. The informed consent represents a collaborative, two-way process between patient and surgeon (12). However, informing patients with temporal lobe epilepsy and baseline memory deficits is uniquely challenging. Variations in levels of health and statistical literacy, and the amount of detail people wish to hear also need to be considered. Data from the semi-structured interviews highlighted what was recalled from the meeting with the surgeon and what was identified as important to the individuals in the current sample.

Prompted by confidence in the consensus decision of a specialized epilepsy team, decisions to proceed with surgery were often made in advance of meeting the neurosurgeon. Signing the consent itself was viewed mostly as a necessary formality but, meeting the surgeon and associated perceptions that they were in the hands of an experienced epilepsy surgeon were vital, and confirmed a sense of caring. Detailed recall of the procedure appeared less important to our study participants than the effective communication of trust. With respect to procedural recall, these findings echoed those of a literature review of 13 internationally conducted studies that addressed the consent process across a range of surgical procedures. The review found that recall and understanding of procedural risks and complications was often low, and especially when information was communicated in traditional verbal ways (21). Making accomodations in the consent process for poor baseline recall is especially relevant in TLE since hippocampal pathology implicates the cognitive and emotional networks of memory and decision-making. One individual suggested that receiving a handout of frequently-asked-questions at the end of the visit, would compensate for a compromised memory. Participants actively listened for confirmation from the surgeon that surgery was a correct and safe treatment approach. They also looked for non-verbal signs that the neurosurgeon was confident about the outcome. Active affirmation from family members present during the clinical visit was simultaneously sought.

Overall, two findings stood out in the current study. Firstly, what was remembered with respect to procedural risks and seizure freedom, was often not accurate, yet participants felt satisfactorily informed. Although participants valued hearing percentages, numerical presentations of risks and benefits were not accurately recalled, and the implications of risks, for example, to memory or visual fields were difficult to imagine. Doubt was reserved due to lack of personal experience with brain surgery and the unknown implications of procedural side-effects or complications. These findings were not unusual, and served to emphasize that anticipating how patients may interpret language and understand the implications is integral to the consent process (11). Recall is only one component of comprehension and treatment decisions made by patients are not necessarily logical or rational (9).

Secondly, personal beliefs, subjective perceptions and expectations weighed strongly in how the consent process was experienced and understood. Once participants agreed that seizure control was unlikely to improve with medicines alone, it was easier for them to accept the surgical option as the definitive treatment. Neurologists who were optimistic about surgical outcomes played a crucial role in encouraging participants to move care forward with their care. Even so, time was required to ponder personal and family implications of surgery, especially the risk of dying from the procedure. Rationalizing fear, and ultimately signing the consent was an emotional event that took courage and faith (9). It was also apparent that the clinical team were not privy to the many intimate reflections that occur in people's minds once the formal step of signing has been taken. Contemplating a changed self after surgery was reflected in our data and has been explored as a common concern of neurosurgical patients (29).

Following data collection for the current study, two (11%) participants declined to proceed with surgery. We suggest a future study to explore perceptions among patients who opted not to proceed with surgery despite having signed a consent. Unfortunately, and to underscore the seriousness of epileptogenic mechanisms, there were two incidents of sudden unexpected death from epilepsy (SUDEP) several months after surgery in just this small sample.

Regarding implications for clinical practice, an enlightening comment was made by one participant drawing attention to her perception of a gap in our approach when she said.

All you hear are the risks and, “This is what we're going to do to you. This is your possibility of cure.” It would have been nice if they said, “And this is what life is going to look like afterwards?” The answer I kept getting was, “It'll be individual. We can't tell you what's going to happen after the surgery.” So, it gave me a sense of even they are wondering how I will react. It's going to be an individual thing.

Thus, anticipating how life might look after surgery is crucial to subjective experience and is especially relevant in situations where the goals of surgery are prophylactic vs. curative. A detailed discussion about life post-resection is generally not part of disclosure. Unfortunately discordant expectations are associated with adjustment difficulties that undermine smooth transistions to wellness (30), highlighting the ethical obligations of the interdisciplinary team in the pre- and post-operative periods. The self-image narrative including personal social worlds is integral to decision-making especially after temporal lobe surgery where the post-surgical results may present unpredictable adjustment difficulties (16). Relevant to the study population, we submit that there is a fine line between subjective perceptions that cannot be controlled by the clinical team, and the ability of individuals to process information. Lack of experience with a given treatment circumstance may lead to risks being over- or-understated (9). One way to overcome misperceptions and information gaps is to conceptualize the practice of informed consent as systematic, structured and progressive with three stages, namely, preparation, signing and follow-up after signing. Such an approach allows time for content to be emotionally processed and clarified. Multiple check-points also enable clinicians to closely evaluate expectations especially in the face of underlying memory and cognitive disability.

## Strengths and Limitations

This qualitative study fills an important knowledge gap with respect to what the process of informed consent for surgery was like for a sample of patients with drug-resistant TLE. The availability of verbal comprehension scores represents an important strength for a qualitative study that addresses treatment decision-making. However, a study limitation is that the sample was drawn from a single, specialized epilepsy center located in a large US city. Sampling bias may also be reflected as the study participants all had sufficient medical insurance to access specialized care. Although the study illicited in-depth, personal insights for a sample of people with drug-reistant TLE, it may not be possible to generalize the findings to those with extra-temporal epilepsies given potentially different surgical risks.

## Conclusions and Future Research

The nuances of the surgical consent serve to calibrate a balanced picture that comprises an exquisitely complex decisional process. While uncontrolled seizures constitute a serious threat to life, and present greater risks than the surgery itself, the method of information delivery should never be coercive. Obtaining an informed consent does not occur at a single point in time, but takes lengthy preparation and counseling, and is a significant human experience in-and-of itself.

Future research that informs consent decisions may help identify better ways to prepare patients for surgery. Additionally, futher study of how best to train the clinical team is warranted and may serve to mitigate the natural hesitation toward elective brain surgery. Such teaching should include how to present inherent trade-offs, and identify what is important to individual patients and families. Establishing a structured, interdisciplinary approach to surgical preparation may address key reasons surgery is underutilized and also support a rehabilitative approach to epilepsy surgery that is accepted as the standard of best practice.

## Data Availability Statement

The datasets presented in this article are not readily available because this is a qualitative study. The data consists of transcripts of the interviews. Requests to access the datasets should be directed to Sandra R. Dewar PhD, APRN, FAES, FAAN; sdewar@mednet.ucla.edu.

## Ethics Statement

Ethical Approval for the study was obtained from the Institutional Review Board at University of California Los Angeles (UCLA) (#18-001356). The patients/participants provided their written informed consent to participate in this study.

## Author Contributions

SD and HP jointly conceived and designed the study, analyzed the data, and drafted the initial manuscript. IF performed all informed consents for surgery and contributed to manuscript discussions and revisions.

## Funding

This work was supported by UCLA intramural funding (#405230-1A-19900-HP).

## Conflict of Interest

The authors declare that the research was conducted in the absence of any commercial or financial relationships that could be construed as a potential conflict of interest.

## Publisher's Note

All claims expressed in this article are solely those of the authors and do not necessarily represent those of their affiliated organizations, or those of the publisher, the editors and the reviewers. Any product that may be evaluated in this article, or claim that may be made by its manufacturer, is not guaranteed or endorsed by the publisher.
